# Thiophene Sulfone Single Crystal as a Reversible Thermoelastic Linear Actuator with an Extended Stroke and Second-Harmonic Generation Switching

**DOI:** 10.1021/jacs.4c17448

**Published:** 2025-02-24

**Authors:** Zhihua Wang, Rongchao Shi, Ibrahim Tahir, Durga Prasad Karothu, Puxin Cheng, Wenqing Han, Liang Li, Yongshen Zheng, Panče Naumov, Jialiang Xu, Xian-He Bu

**Affiliations:** † School of Materials Science and Engineering, Tianjin Key Laboratory of Metal and Molecular Materials Chemistry, Frontiers Science Center for New Organic Matter, Nankai University, Tongyan Road 38, Tianjin 300350, P. R. China; ‡ Smart Materials Lab, New York University Abu Dhabi, P.O. Box 129188 Abu Dhabi, UAE; § SINOPEC (Beijing) Research Institute of Chemical Industry Co., Ltd. Yanshan Branch, Fenghuangting Road 15, Beijing 102500, P. R. China; ∥ Novel Materials Development Lab, Sorbonne University Abu Dhabi, P.O. Box 38044 Abu Dhabi, UAE; ⊥ Center for Smart Engineering Materials, New York University Abu Dhabi, P.O. Box 129188 Abu Dhabi, UAE; # Research Center for Environment and Materials, Macedonian Academy of Sciences and Arts, Bul. Krste Misirkov 2, MK-1000 Skopje, Macedonia; ¶ Molecular Design Institute, Department of Chemistry, New York University, 100 Washington Square East, New York, New York 10003, United States

## Abstract

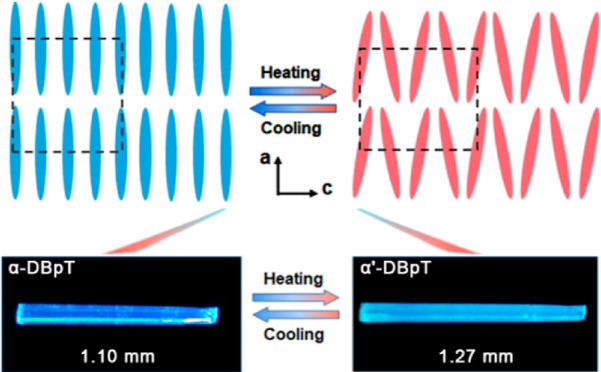

Dynamic organic crystals are becoming recognized as some of the fastest materials for converting light or heat to mechanical work. The degree of deformation and the response time of any actuating material are often exclusive of each other; however, both factors influence the material’s overall performance limits. Unlike polymers, whose disordered structures are not conducive to rapid energy transfer, cooperative phase transitions in dynamic molecular crystals that are amenable to rapid and concerted martensitic-like structure switching could help circumvent that limitation. Here, we report that single crystals of a dibenzothiophene sulfone derivative exhibit extraordinarily large, rapid, and reversible elongation when they undergo a thermally induced phase transition. The value for the linear stroke of ∼15% along the long crystal axis with retention of macroscopic integrity of this material is remarkable and capitalizes on an anisotropic lattice switching with relative changes of 14.8% and −9.5% along its crystallographic *a* and *c* axes, respectively, resulting in a visible macroscopic elongation of the crystal. The transitioning crystals deliver forces ranging from 0.19 to 15 μN and a work density of ∼7 × 10^–3^ J m^–3^. The phase transformation is accompanied by a change in symmetry between centrosymmetric and noncentrosymmetric space groups and a significant change in both the fluorescence and the second-order nonlinear optical (NLO) response. The combination of these properties makes this material a favorable choice for low-power, precise, and small-scale NLO actuation applications.

## Introduction

The growing interest in dynamic systems has increased the search for materials that offer fast and reversible response, efficient energy conversion, and low-to-none fatigue in operation.^1−9^ Among the available classes of “smart” materials, the dynamic crystals are distinct in leveraging the cooperative molecular motions within the crystal and their amplification both in space and over time, the process being facilitated by their long-range ordered and densely packed structures.^5,10−15^ The mechanical flexibility associated with the softness of intermolecular interactions enable dynamic molecular crystals to maintain their macroscopic integrity even after experiencing significant, rapid, and observable reshaping or motion, and this asset is thought to present opportunities for harvesting, transduction, and conversion of energy.^16−24^ The opportunity for thermal stimulation of molecular crystals that exhibit significant macroscopic deformations warrants special attention from the perspective of their utility in devices such as thermal actuators and expansion compensators. Moreover, they are essential as active components in mechanically adjustable elements for actuation and energy harvesting, including applications in microfluidic valves and dynamic components in soft robotics.^1,5,17,25−29^ However, the slow mechanical response and limited stroke range observed with most of these materials pose significant challenges that currently hinder their direct implementation in micro- and macro-devices.^30^

The goal of achieving strong thermal deformation alongside high energy density has driven significant research efforts focused on identifying crystals with diverse flexible structures. For instance, metal–organic framework crystals with a “wine-rack” (“lab jack”)-like topology have been reported to undergo significant deformation of their rhomboid-shaped frameworks that normally results in considerable anisotropic thermal expansion in the direction that allows for translational movement.^31−35^ Collective reorientation of nonspherical molecules induced by molecular rotation has been demonstrated as another effective approach to achieve extraordinary thermal deformation of molecular crystals.^7,13,14,16,25,27,36−38^ To address the molecular interactions and steric hindrance that restrict molecular redirection in crystalline materials, a strategy called “entropy increase” was developed^6,19,27,39−41^ that relies on special entropy-reservoir units, such as circular molecules, which can overcome energy barriers by gaining entropy from the molecular thermodynamics. Recently, we reported a series of thermally activable molecular crystals of fluorenone derivatives that combine rigid conjugate planes with rotatable phenyl rings and exhibit significant thermal deformations while delivering considerable work densities.^5,28,42,43^ However, these crystals consistently grow in parallelepiped shapes and usually expand comparably in two directions, resulting in a limited relative expansion (stroke) along each direction. In this study, we report a thermoelastic crystalline material based on dibenzothiophene-5,5-dioxide that undergoes a thermally induced single-crystal-to-single-crystal (SCSC) phase transition. The phase transition causes the rod-like microcrystals of the material to elongate primarily along their longest side, resulting in work density that is nearly twice that of the previously reported fluorenone-based dynamic crystals. This material does not only fill the existing knowledge gap by displaying both fairly reversible structural changes and exceptional dynamic performance upon thermal stimulation but it also sets the basis to explore the desirable extremes in the intricate structure–activity relationships between microstructural perturbations and mechanical response.^3,29,44,45^

## Results and Discussion

**Figure 1:**
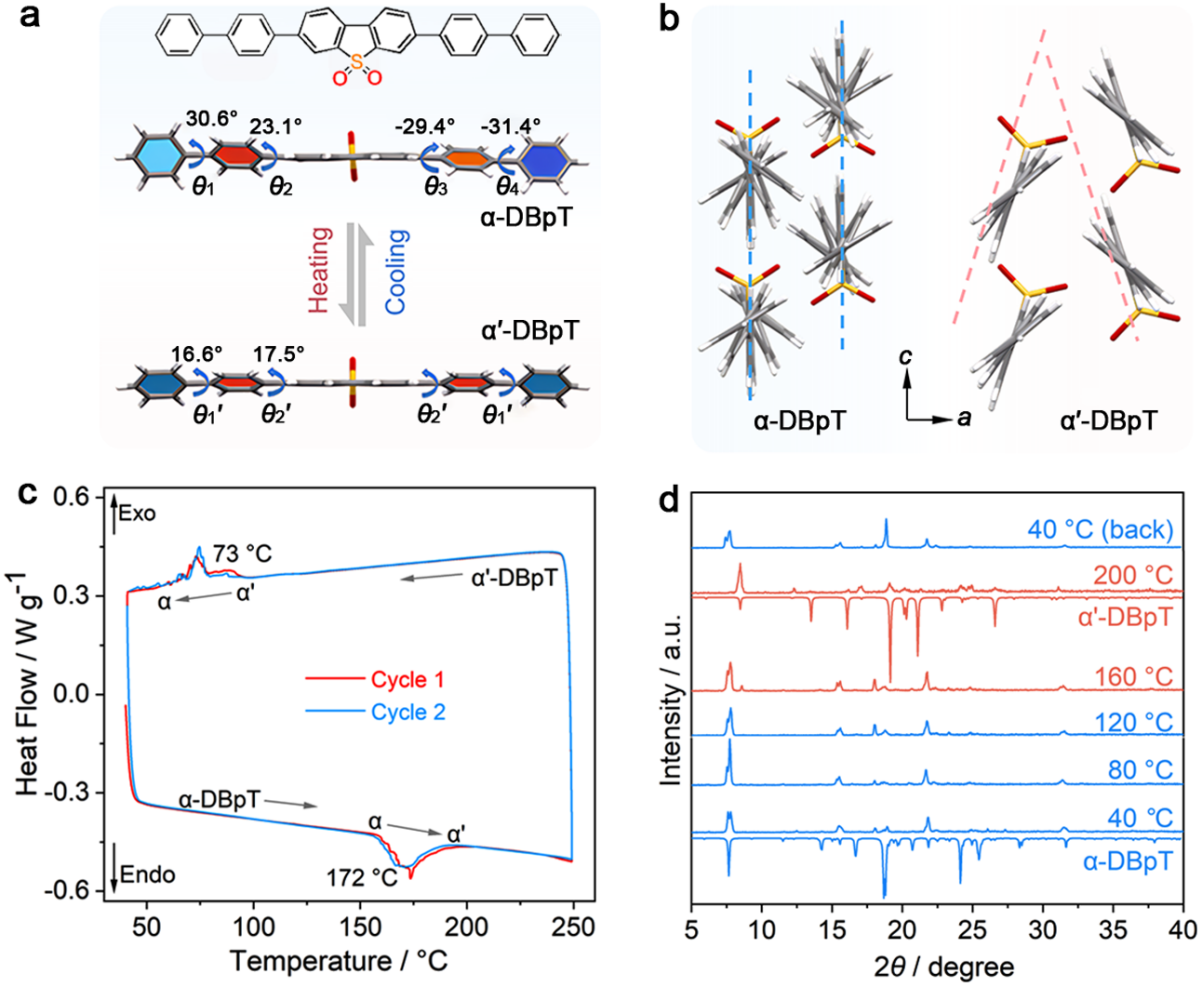
Structure and thermally induced structural phase transition of DBpT. (a) Thermally induced changes in the intramolecular dihedral angles θ_1_ – θ_4_ between the phenyl rings in the structures of phases α and α′. (b) Comparison of the molecular packing in the two phases. (c) DSC diagrams showing the thermal effects upon transitioning between phases α and α′ over two consecutive thermal cycles. (d) Experimental VT-PXRD patterns of α-DBpT compared to the simulated patterns of phases α and α′.

The compound, 3,7-di([1,1′-biphenyl]-4-yl)dibenzo[*b*,*d*]thiophene 5,5-dioxide (DBpT), was synthesized by Suzuki coupling between 3,7-dibromodibenzothiophene sulfone and 4-biphenylboronic acid (Figure S1).^46^ It was found to crystallize as two polymorphs, hereafter referred to as α-DBpT and β-DBpT, obtained by using the solvothermal method in toluene and tetrahydrofuran, respectively. Additionally, a new phase, α′-DBpT, can be obtained by in situ heating of α-DBpT. α-DBpT crystallizes in the monoclinic space group *P*2_1_/*c*, with unit cell parameters *a* = 7.3069(3) Å, *b* = 28.8319(10) Å, *c* = 12.1968(4) Å, β = 90.372(2)°, and *Z* = 4 (Table S2). Due to the steric hindrance, the DBpT molecule in the crystal of the α phase is twisted along its length, as reflected in dihedral angles between the phenyl rings θ_1_ – θ_4_ = 30.6°, 23.1°, −29.4°, and −31.4°, as defined in Figure 1a. The molecules are packed along the crystallographic *a* axis and interact with each other via intermolecular π···π interactions. Furthermore, multiple weak intermolecular C–H···π interactions between the molecules along the crystallographic *a* and *c* axes could contribute to the ability of the structure to absorb stress caused by minor molecular alterations. These interactions, apparent from the two-dimensional fingerprint plots of the Hirshfeld surfaces (Figure S2), could also be the reason for the sustained integrity of the lattice during the phase transition (see below).^47^ β-DBpT crystallizes in the space group *P*2_1_/*n*, with unit cell parameters *a* = 9.4972(1) Å, *b* = 9.3172(1) Å, *c* = 28.5942(4) Å, β = 95.463(1)°, and *Z* = 4. With dihedral angles θ_1_ – θ_4_ = 27.5°, 12.3°, 24.5°, and 12.0° (Figure S3), the overall twisting of the molecule in the β phase is smaller than that in the α phase, and thus the molecule is flattened on average, resulting in weaker C–H···π interactions (Figure S4).

Thermogravimetric analysis (TGA) showed that the crystals of both α-DBpT and β-DBpT are thermally stable below their melting temperatures 350 and 340 °C for α-DBpT and β-DBpT, respectively (Figure S5). The TGA curve of the β-phase shows a weight loss around 250 °C due to evaporation of the residual solvent, which disrupts the crystal structure and results in increased opacity of the crystal surface and enhanced fluorescence (Figure S6). Moreover, in situ single-crystal X-ray diffraction (SCXRD) analysis revealed that the structure of the high-temperature phase (α′-DBpT), obtained via an SCSC phase transition from form α-DBpT (for details, see below), is different from that of form β-DBpT. In this high-temperature phase, the main dihedral angles in the DBpT molecule decrease relative to those in the low-temperature phase, and the molecule acquires symmetry (Figure 1a). The two phenyl rings adjacent to the central plane have an identical dihedral angle θ_2_′ = 17.5°, whereas the benzene rings at both ends of the molecule are at an angle θ_1_′ = 16.6° relative to the plane of the central dibenzothiophene fragment. Moreover, the arrangement of the neighboring molecules in the α′-DBpT phase changes from parallel to bent with a dihedral angle of 37.0° (Figure 1b). This change in intramolecular dihedral angles affects the steric requirements for interaction with the neighboring molecules, which, in turn, promotes overlap between adjacent molecules along the crystallographic *c* axis and results in shrinking along that axis. Detailed thermal analysis using differential scanning calorimetry (DSC) confirmed that, when heated, α-DBpT undergoes a phase transition in the temperature range 155–195 °C (Δ*H* = 4.7 × 10^3^ J mol^–1^, Δ*S* = 10.5 J mol^–1^ K^–1^; Figure 1c). The reverse phase transition occurs at 100–60 °C on cooling, with latent heat and an undercooling characteristic for a first-order phase transition.^47^ In analysis by variable-temperature powder X-ray diffraction (VT-PXRD), the characteristic diffraction peaks of α-DBpT at approximately 7.6° and 15.5° disappear at 200 °C, and new diffraction peaks emerge around 8.4° and 13.5° (Figure 1d). This observation is consistent with the thermally induced phase transition detected by DSC. Upon cooling to ∼40 °C, the Bragg diffraction peaks essentially return to their positions before heating (40 °C), lending additional support for the reversibility of the phase transition.

**Figure 2:**
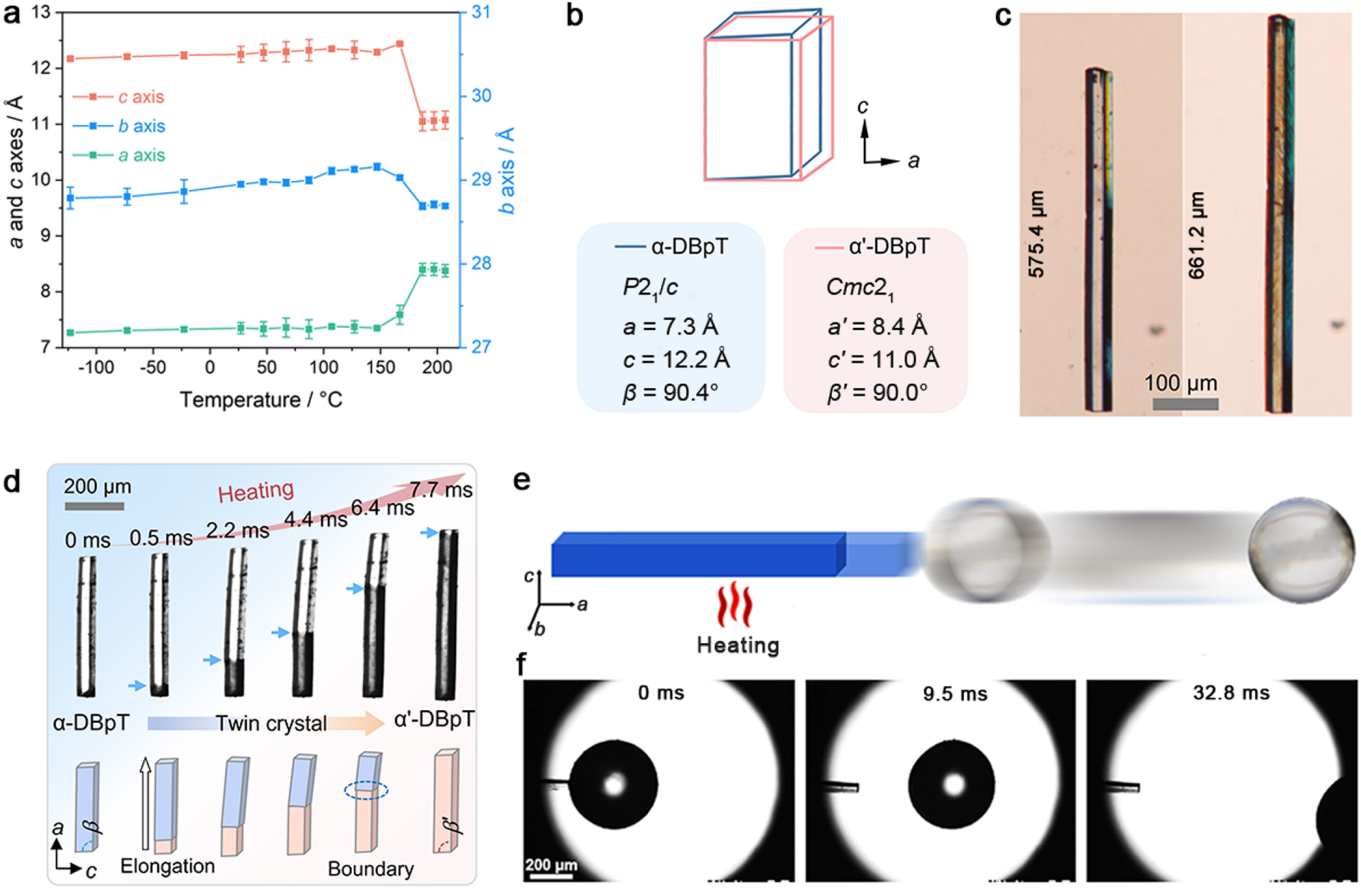
Changes in crystal appearance and structure that occur upon phase transition between phases α and α′. (a) Variation in the lengths of the unit cell axes with temperature within the range from −123 to 207 °C. (b) Schematic diagram showing overlapped representation of the unit cell before and after the phase transition. (c) Optical images showing, under identical magnification, the dramatic change in the length of the crystal of DBpT caused by the phase transition from form α to form α′. (d) Elongation of a crystal of α-DBpT captured by a high-speed camera, showing the phase transition propagation from one end of the crystal to the other. (e,f) Schematic diagram (e) and snapshots (f) of a crystal of α-DBpT that elongates and pushes a glass ball, demonstrating the capacity of the transitioning crystal as a single-stroke actuator.

In situ VT-SCXRD analysis suggests that the unit cell parameters of α-DBpT remain nearly unchanged below 160 °C (Figure 2a). Above this temperature, a sudden change in the unit cell corresponds to the transition to α′-DBpT. Specifically, while the *b* axis remains almost unchanged (−0.48%), the *a* and *c* axes undergo strong and converse changes of +14.8% and −9.5%, respectively (Figure 2a). As a result of the synergistic changes in the intermolecular interactions and the packing, α′-DBpT has a lower symmetry compared to that of α-DBpT, and it was modeled in the orthorhombic space group *Cmc*2_1_, with unit cell parameters *a* = 28.693(9) Å, *b* = 11.038(3) Å, *c* = 8.375(2) Å, and *Z* = 4 (Figure 2b). The alteration of the unit cell induces a concomitant change in the size of the crystal, which manifests as dramatic macroscopic elongation of about 15% (Figure 2c, Table S1, and Movie S1). The thermal elongation is reversible, and the crystal shrinks as the temperature is slowly decreased over the phase transition (Figure S7). However, internal defects and inherent brittleness result in partial disintegration.

Detailed analysis of the crystal transformation by using a high-speed camera shows that this expansion is due to very rapid propagation of the interphase front from one end of the crystal to the other (Figure 2d and Movie S2), indicating possible cooperativity in the structure switching.^19,48^ As shown in Figure 2d, the phase transition is initiated at the end that had noticeable defects and rapidly propagates along the crystal’s long axis, creating a distinct boundary between the domains of the two phases.^5,19^ Concurrently with its transition from the monoclinic to the orthorhombic structure, the crystal bends very slightly (2–4°) at the phase boundary (Movie S2). This sizable thermally induced elongation confers the potential of the crystals of DBpT as microactuation devices that can actuate other objects (Figure 2e). To demonstrate this capability, one end of a single crystal (∼0.5 mm in length) was affixed to a glass surface, and a free-standing glass ball (∼0.25 μg in weight) was placed in contact to its other (free) end (Figure 2f).^5,17^ Upon heating to 165 °C, the crystal underwent instantaneous elongation due to the SCSC transition to the α′ phase and displaced the glass ball by around 1.1 mm within 32.8 ms (Figure 2f and Movie S3).

**Figure 3:**
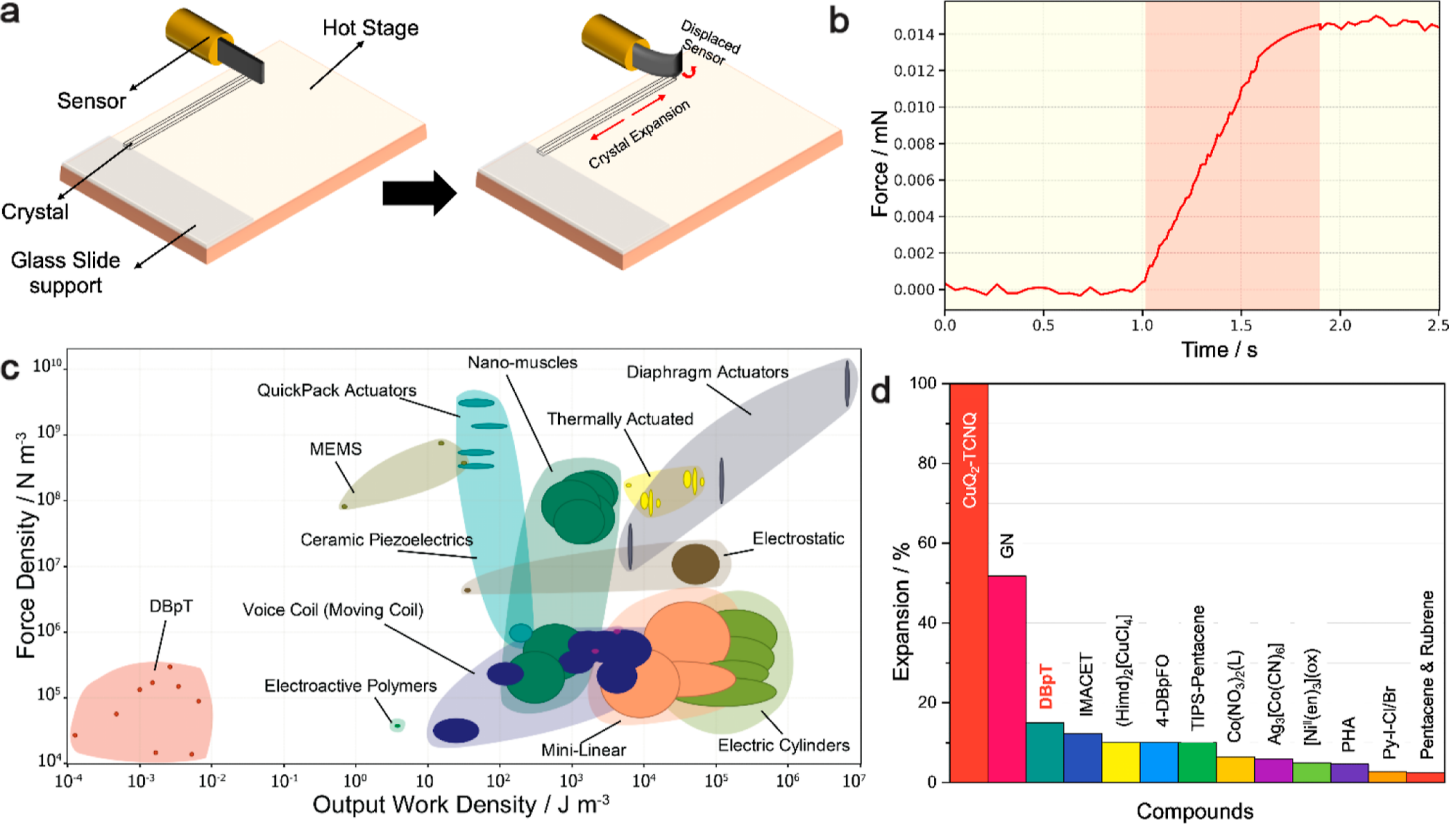
Actuation behavior and performance comparison of α-DBpT crystals. (a) Schematic illustration of the experimental setup for measuring crystal actuation. (b) Force-time graph showing the force generated by a α-DBpT crystal during its phase transition from form α to form α′. The shaded region indicates the phase transition period, highlighting the increase in force exerted on the sensor by the crystal expansion. (c) Ashby plot showing the relationship between the work density and force density for α-DBpT and other actuation materials. (d) Bar chart comparing the expansion percentages of α-DBpT crystals with other thermosalient compounds, demonstrating the comparable expansion performance of α′-DBpT.

To further quantify the actuation forces generated during the phase transition, direct force measurements were performed by using a sensitive force sensor, as illustrated in Figure 3a.^45^ In this setup, each of the studied crystals was, in turn, oriented perpendicular to its axis of expansion, with one end firmly supported against a rigid wall and the other being placed in contact with the force sensor. This arrangement enables precise recording of the force exerted by the expanding crystal during its phase transition until saturation, ensuring maximum force capture. The experimental setup was designed to ensure proper contact between the crystal and the sensor for efficient transfer of momentum. The force sensor was carefully pushed against the crystal, which was supported by the glass slide, and the initial displacement of the sensor was set to a value slightly below zero to confirm contact. This ensures consistent contact during the measurements, and thus the force densities are unaffected by the physical contact. As shown in Figure 3b, the force-time relationship during the transition from form α to form α′ displays notable increase in force, highlighting the phase transition and subsequent actuation. In a series of measurements, forces ranging from 0.19 to 15 μN were recorded for nine tested crystals (the details are provided in Table S3). Measuring the force generated during repeated cycles, however, presented significant challenges, since, as mentioned above, the crystals begin to show fractures, and these fractures become more pronounced with each subsequent cycle.

With these results in hand, the highest work density of DBpT crystals was estimated to be ∼7 × 10^–3^ J m^–3^. This value of the work density is notably lower than other actuating systems reported previously.^24^ We attribute these differences both to the factors that pertain to the nature of stimulus and energy conversion (light vs heat) and to the very different design of the experimental setups (indirect vs direct measurement) used to measure these values, where different setups may be better suited for quantification of specific ranges of the exerted force. To benchmark the performance of the DBpT crystals and identify suitable applications, their force density and work density were compared to those of other common actuators, such as electroactive polymers, voice coils, electric cylinders, and mini-linear actuators (Figure 3c). This comparison helps to evaluate the efficiency of force generation relative to volume and the capacity to convert thermal energy into mechanical work. Although DBpT crystals generate force efficiently, their work density remains comparatively lower, positioning them as a suitable material for low-power, precision applications like microactuators, soft robots, or wearable devices.^24^ Their energy efficiency and fine control capabilities also enhance mechanical safety, making them ideal for applications with power constraints or those requiring careful human interaction. In terms of expansion, the relative expansions or strokes measured for DBpT crystals range from 4.3 to 89 μm, as detailed in Table S3. Figure 3d shows that the impressive 15% expansion of DBpT crystals is much higher compared to other thermosalient compounds reported previously,^5,49−51^ reinforcing their potential for precise, small-scale actuation applications.

During the phase transition of the crystal of α-DBpT, there is a significant change in the molecular stacking mode within the *h*0*l* plane. The distance of the weak intermolecular interactions (C–H···π interactions) in the *h*0*l* plane is approximately 2.8 Å, which is smaller than the sum of van der Waals radii (Figure S8a,b). A similar nonbonding effect has been observed in the thermally stimulated dynamic molecular crystals of two fluorenone derivatives (2.9 Å), 4-DBpFO and 4-DTpFO.^5,28,42^ The phase transitions of these crystals are accompanied by notable changes in the stacking mode of the molecules in the *h*0*l* plane, resulting in anisotropic thermal expansion along the crystallographic *a* and *c* axes. However, the distance of the nonbonding interactions in the *h*0*l* plane of a crystal of β-DBpT exceeds 3.1 Å, which is greater than the sum of the van der Waals radii. Upon thermal stimulation, there is no significant alteration observed in the molecular stacking mode in the *h*0*l* plane and no significant anisotropic thermal expansion in the crystal axes (Figure S8c,d). We conclude that the weak interactions (C–H···π interactions) play an important role in the structural evolution of such dynamic crystals with rigid planes and flexible phenyl side chains. The rearrangement of the phenyl rings serves as a basis for regulation of the weak intermolecular interactions and rearranging of molecular stacking. This goes along with the qualitative hypothesis that the weak intermolecular interactions effectively absorb the stress generated during the structural adjustment, thereby maintaining the macroscopic integrity of the crystal.

**Figure 4:**
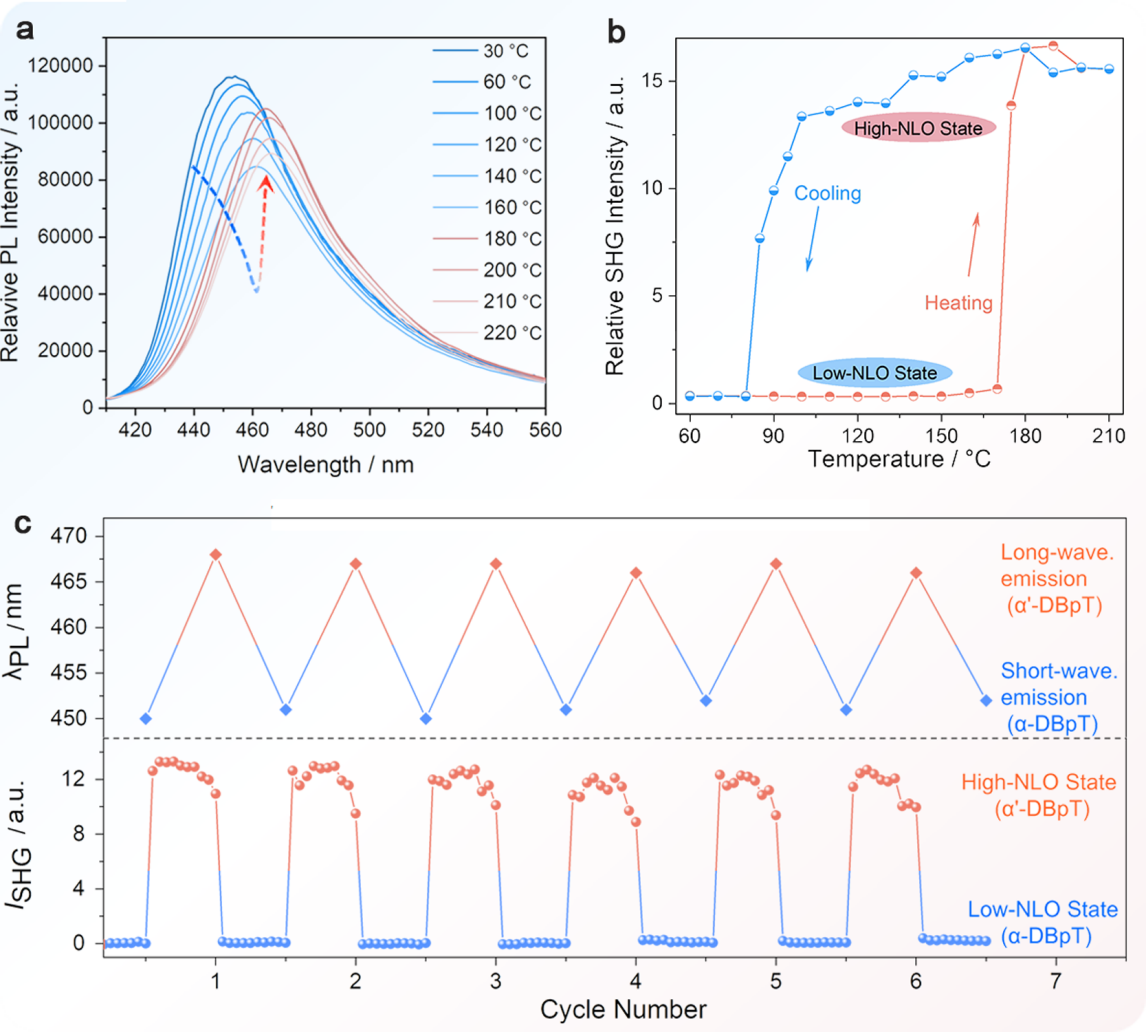
Linear and nonlinear optical properties. (a) Temperature-dependent fluorescence spectra of α-DBpT. The arrow highlights the change in the fluorescence on heating. (b) Temperature-dependent second harmonic generation (SHG) intensity of α-DBpT. (c) Reversibility of the temperature dependence of the linear fluorescence emission and the SHG intensity.

The very different molecular configurations and packing modes of DBpT molecules in phases α and β result in distinctly different optical properties. Under 365 nm excitation, powders of α-DBpT and β-DBpT exhibit fluorescence with emission peaks at 451 and 480 nm, respectively. Interestingly, β-DBpT displays bright cyan light emission only at its edges, probably due to the parallel orientation of the molecular transition dipole moment with respect to the molecular long axis, which is perpendicular to the plane of the lamellar crystal (Figure S9).^52^ The powders of α-DBpT and β-DBpT show high photoluminescence quantum yields of 68.2% and 89.3%, respectively (Figure S10). The conformational changes induced by the increased temperature lead to shifts in the emission in the fluorescence emission spectrum of the α-DBpT crystal, resulting in the observed color change. Upon heating, the photoluminescence intensity of the α-DBpT crystal first decreases gradually but undergoes a sudden increase at 180 °C, a result that corresponds to the phase transition to α′-DBpT (Figure 4a). The normalized fluorescence spectra indicate a sharp redshift during this temperature range (Figure S11). Crystalline powder of α-DBpT displays reversible fluorescence emission changes, with notable fatigue resistance, as inferred by the emission intensity, which remains unaffected even after multiple thermal cycles (Figure 4c).

Crystalline materials capable of reversible conversion of nonlinear optical (NLO) effects in different states have garnered significant interest in the rapidly evolving field of optoelectronic devices.^44,53,54^ The phase transformation of α-DBpT results in a drastic change from a centrosymmetric space group (*P*2_1_/*c*) to a noncentrosymmetric space group (*Cmc*2_1_), leading to a significant change in its second-order NLO response, and therefore, it should result in evolution of second-harmonic generation (SHG). To investigate this NLO switching, we used an NLO testing system equipped with a hot stage to monitor the SHG response. When the temperature surpasses 160 °C, the SHG intensity of the crystal increases rapidly and subsequently stabilizes above 180 °C (Figure 4b). In accord with the DSC results, the SHG intensity of the crystal decreases upon cooling to 90 °C. The crystal displays switching ratio of over 10 in its SHG response,^54^ and this SHG switching effect is maintained even after six cycles (Figure 4c). The reversibility of linear and NLO function broadens the application prospects of DBpT with possible implications for the fields of optical information storage and encryption.^55−57^

## Conclusions

In summary, we present a thermally responsive molecular crystal that exhibits both structural and optical reversibility. Crystals of one of its low-temperature forms show remarkable structural flexibility, leading to significant macroscopic elongation driven by a thermally induced SCSC phase transition. The retention of macroscopic integrity, the impressive 15% expansion, energy efficiency, lower work density, and the opportunity to control the size of these crystals make this material a favorable choice for prototypical low-power precision applications such as microactuators, soft robotics, and wearable devices. Moreover, the thermally induced rearrangement of the phenyl rings facilitates molecular reconfiguration, offering a promising model for the development of similar dynamic materials that are capable of rapid, reproducible, and controllable shape deformations.

## Supporting Information Available

Experimental procedures, synthetic route to DBpT, 2D plots of the Hirshfeld analysis, distribution of intermolecular interactions, TGA of α-DBpT and β-DBpT, morphology and crystal structures, photoluminescence spectra and quantum yields, linear expansion of α-DBpT crystals, crystallographic and refinement data for DBpT, and summary of the data collected for nine individual crystals using the AE801 force sensor (PDF)
Elongation of an α-DBpT crystal observed by a light microscope (MP4)
Elongation of an α-DBpT crystal recorded with a high-speed camera (MP4)
Crystal that elongates and propels a glass sphere (MP4)
