# Supplementary material for: Thiophene Sulfone Single Crystal as a Reversible Thermoelastic Linear Actuator with an Extended Stroke and Second-Harmonic Generation Switching
Source: J Am Chem Soc. 2025 Feb 24;147(9):7749–56. doi: 10.1021/jacs.4c17448 (PMC11887050; doi:10.1021/jacs.4c17448)
Supplement: Supplementary file 1 — ja4c17448_si_001.pdf [file ja4c17448_si_001.pdf]

SUPPORTING INFORMATION

Thiophene Sulfone Single Crystal as a Reversible Thermoelastic Linear Actuator with an Extended Stroke and Second-Harmonic Generation Switching

Zhihua Wang,[†,∇] Rongchao Shi,[§,∇] Ibrahim Tahir,[‡,∇] Durga Prasad Karothu,[‡] Puxin Cheng,[†] Wenqing Han,[†] Liang Li,[‡,ǁ] Yongshen Zheng,[†] Panče Naumov,*[,‡,⊥,#,¶] Jialiang Xu,*[,†] Xian-He Bu[†]

[†]School of Materials Science and Engineering, Tianjin Key Laboratory of Metal and Molecular Materials Chemistry, Frontiers Science Center for New Organic Matter, Nankai University, Tianjin 300350, P. R. China

[§]SINOPEC (Beijing) Research Institute of Chemical Industry Co., Ltd. Yanshan Branch, Beijing 102500, P. R. China

[‡]Smart Materials Lab, New York University Abu Dhabi, P.O. Box 129188, Abu Dhabi, UAE

[ǁ]Novel Materials Development Lab, Sorbonne University Abu Dhabi, P.O. Box 38044, Abu Dhabi, UAE

[⊥]Center for Smart Engineering Materials, New York University Abu Dhabi, P.O. Box 129188, Abu Dhabi, UAE

[#]Research Center for Environment and Materials, Macedonian Academy of Sciences and Arts, Bul. Krste Misirkov 2, MK–1000 Skopje, Macedonia

[¶]Molecular Design Institute, Department of Chemistry, New York University, 100 Washington Square East, New York, New York 10003, United States

[∇]These authors contributed equally to this work

*Corresponding authors: Panče Naumov (pance.naumov@nyu.edu); Jialiang Xu (jialiang.xu@nankai.edu.cn)

This PDF file includes:

Methods
Supporting figures S1 to S11
Supporting tables S1 to S3
Supporting references
Legends for the supporting movies S1 to S3

1. Methods

Synthesis of DBpT. Under argon atmosphere, $Pd(PPh_3)_4$ (58.0 mg, 0.05 mmol), 3,7-dibromodibenzothiophene sulfone (520.1 mg, 1.4 mmol), and 4-biphenylboric acid (673.3 mg, 3.4 mmol) were added to a mixture of toluene (100.0 mL) and K_2CO_3 (20.0 mL, 2.0 M), and the reaction mixture was refluxed overnight. After cooling and removing most of the solvent in vacuum, the solid mixture was mixed with deionized water (50.0 mL × 3), washed, and filtered to remove K_2CO_3. The solid was collected and placed in a 250 mL single-mouthed bottle containing 100.0 mL of ethanol for 5 hours to remove the excess 4-biphenylboric acid. After filtration and drying, 463.4 mg of white powder was obtained with a yield of 89%. Anal. calcd. for $C_{36}H_{24}O_2S$: C 91.71, H 4.99; found: C 91.90, H 4.58. ^1H NMR (400 MHz, Chloroform-d): δ (ppm) 8.12 (s, 2H) 7.93 (q, J = 7.9 Hz, 4H), 7.75 (m, 12H), 7.49 (t, J = 7.5 Hz, 4H), 7.4 (d, J = 7.4 Hz, 2H).

Single crystal preparation. To prepare crystals of α-DBpT, 10.0 mg DBpT was mixed with 5.0 mL of toluene, and the resulting mixture was heated to 180 °C under solvothermal conditions for 10 hours. The mixture was then allowed to cool to room temperature within 24 hours. This process resulted in the formation of well-defined rod-shaped microcrystals of lengths ranging from 100 to 500 μm, which were suitable for analysis with single-crystal X-ray diffraction. Crystals of β-DBpT were obtained in the same way as the α-DBpT crystals, except that the solvent used was tetrahydrofuran.

X-ray diffraction analysis. Variable-temperature SCXRD data of α-DBpT were collected on Rigaku SuperNova CCD diffractometer with CuK_α radiation (λ = 1.5418 Å) and the multiscan mode. The structures were solved by direct methods using the SHELXS program within the Olex2 software and refined by the full-matrix least-squares method with SHELX.[1,2] All non-hydrogen atoms were refined by anisotropic displacement parameters, while the hydrogen atoms attached to carbon atoms were calculated in ideal positions. Crystallographic data for structures reported in this paper have been deposited in the Cambridge Crystallographic Data Centre under reference numbers CCDC 2286599 to 2286601 (Table S2). These data can be obtained free of charge from www.ccdc.cam.ac.uk/data_request/cif. The variable-temperature powder XRD data of the crystal from room temperature to 200 °C were recorded on the MiniFlex600 powder X-ray diffractometer.

Microscopy. The fluorescence images were acquired with a LEI-TECH LK40FL microscope. The optical microscopic images were obtained with a LK40POL optical microscope. To capture images of the changes in crystal shape, the microscope was fitted with a Linkam THMS600 thermal stage, operated at a heating rate of 10 K min^{-1}. In order

to record the details of the crystal elongation and the dynamic response time, a Revealer high-speed camera (M230) at a frame rate of 4500 fps was used.

Measurement of crystal actuation force. The force exerted by α-DBpT crystals during their transition to form α′ was measured using an AE801 force sensor. This sensor operates on a cantilever mechanism and is constructed from a single crystal of n-type silicon positioned between ion-implanted p-type resistors (details on the design and operating principles are provided in ref. [3]). When mechanical stress deflects the sensor's beam, it induces a piezoresistive effect, leading to a corresponding change in output voltage. To calibrate the sensor, incremental deflections of 10 μm were applied, maintaining a linear relationship between deflection and voltage output. The conversion from voltage to deflection was then adjusted, and Equation 1 was applied to calculate the force generated by deflection,

$$\delta = \frac{Fs^2}{3EI} \qquad (1)$$

where δ is the maximum cantilever tip deflection, F is the applied force, s is the length of the cantilever, and E is the shaft's Young's modulus. For the force sensor used here, s = 5 mm, E = 160 GPa, and I = $2.67 \cdot 10^{-16}$ m^4. For measurement, form α crystal was carefully sandwiched between a rigid wall and the tip of the force sensor below the phase transition temperature. Then the temperature was increased up to 180 °C to ensure complete phase transition. The cantilever deflection was recorded during the phase transition which recorded the force generated by the crystal's elongation. The validity of the results was assured by following a three-stage protocol. First, as an initial system check, before conducting any measurements, we performed thorough checks of the setup with and without crystals to rule out any erroneous or faulty issues with the sensors, the setup, or the software. This ensures that the system is functioning correctly and is free from artifacts that could affect the measurements. Second, the setup was validated by performing force measurements on known crystals whose work densities have been independently reported in earlier studies. By comparing the results with the previously reported values, we confirmed that our setup is capable of providing consistent measurements. Only after observing these, we proceeded to measure the force generated by the crystals in the current study. Third, the reproducibility and consistency were checked by performing force measurements on multiple crystals of the material under study to ensure reproducibility and consistency in the results. The pronounced difference in work densities with some previous results can be attributed to a combination of factors, including slower kinetics and energy dissipation in thermally stimulated systems compared to photomechanical systems, the relatively fragile nature and the occurrence of fracturing of the crystals which limits force generation, the use of rigid piezoelectric

sensors, and potential inefficiencies in the phase transition, such as incomplete transitions during force measurements.

Actuator data and materials property plots. The actuator data was retrieved and plotted from the CES Selector 2019, Granta Design Limited, Cambridge, UK, 2019.[4] The data of other actuating materials in the database are divided into over 20 classes depending on the available attributes.

2. Supporting figures

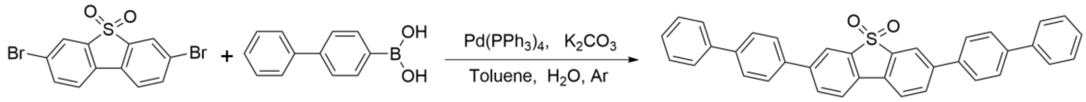

Figure S1. Synthetic route to DBpT.

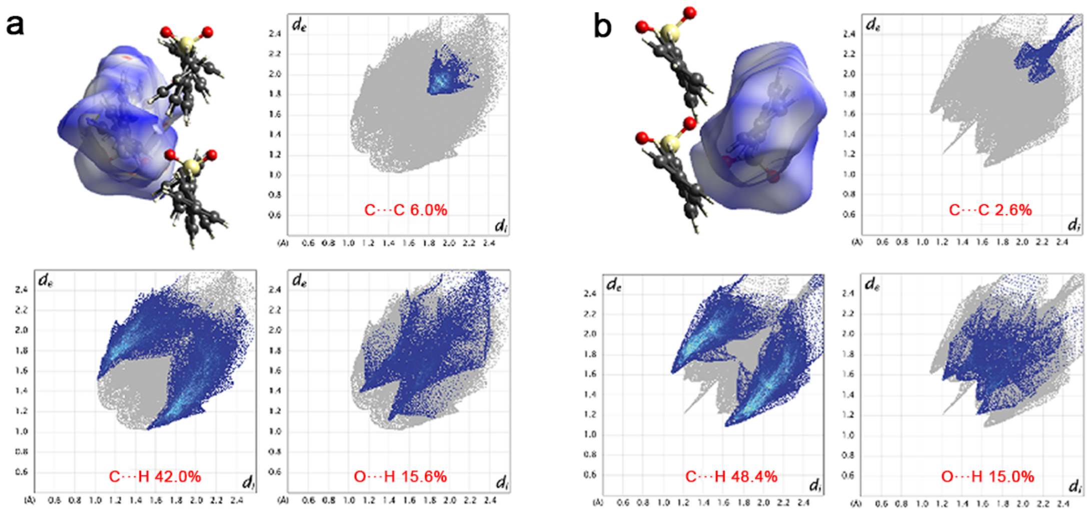

Figure S2. Two-dimensional plots of the Hirshfeld analysis of (a) α-DBpT and (b) α′-DBpT.

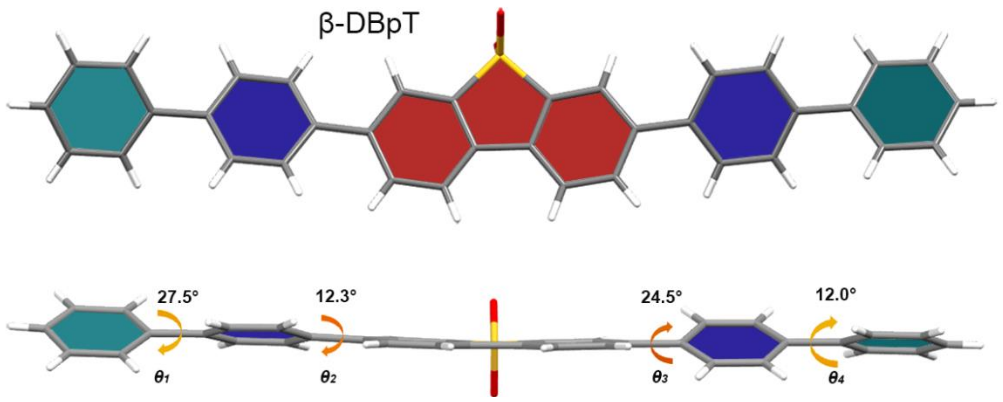

Figure S3. Definition of the intramolecular dihedral angles θ_1 - θ_4 and their values in the crystal of β-DBpT.

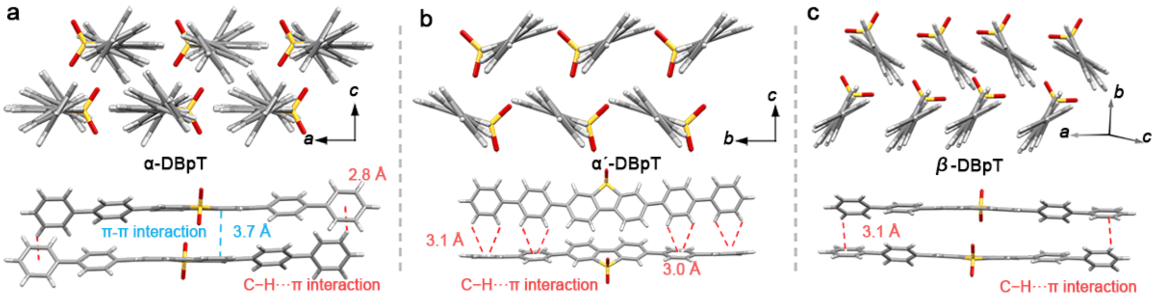

Figure S4. Distribution of intermolecular interactions in (a) α-DBpT, (b) α′-DBpT and (c) β-DBpT.

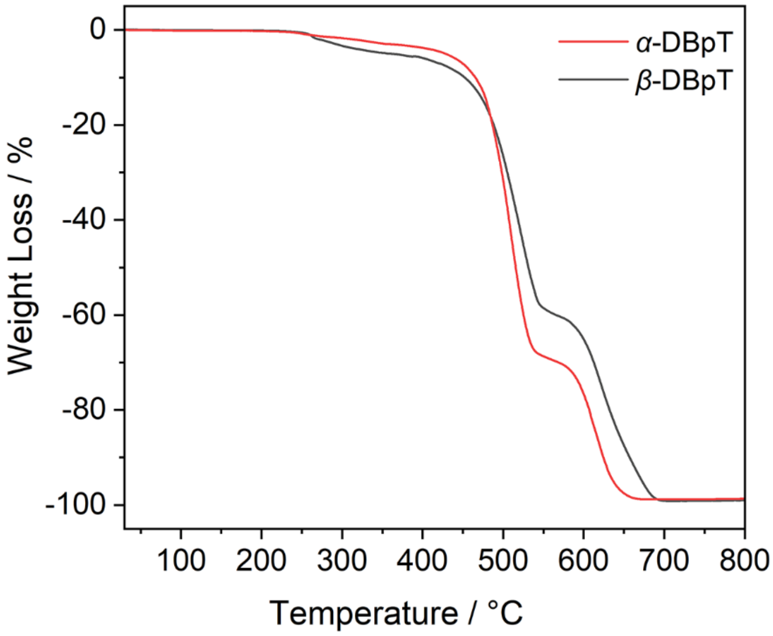

Figure S5. Thermogravimetric analysis (TGA) of α-DBpT and β-DBpT.

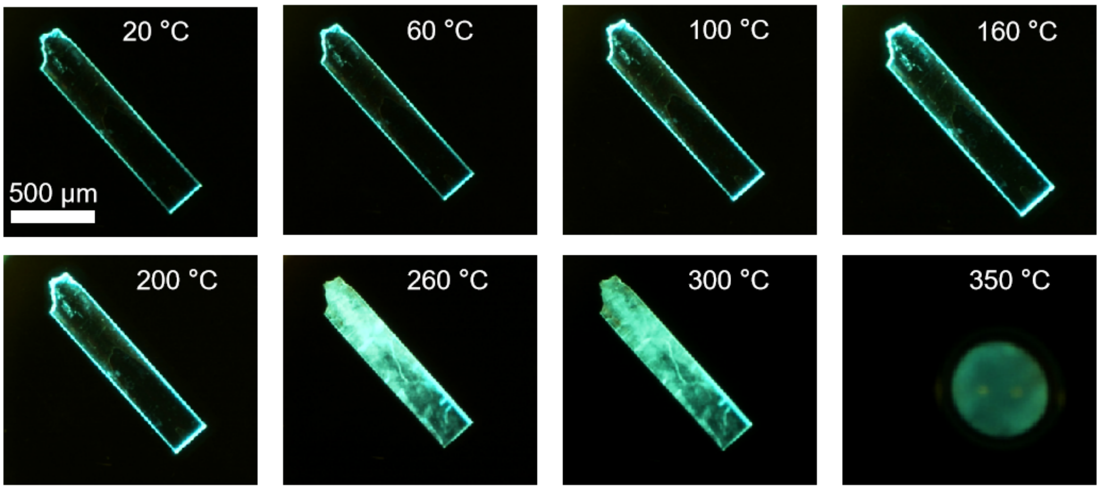

Figure S6. Photographs of a β-DBpT crystal that was heated and observed with a fluorescence microscope (excitation wavelength: 365 nm).

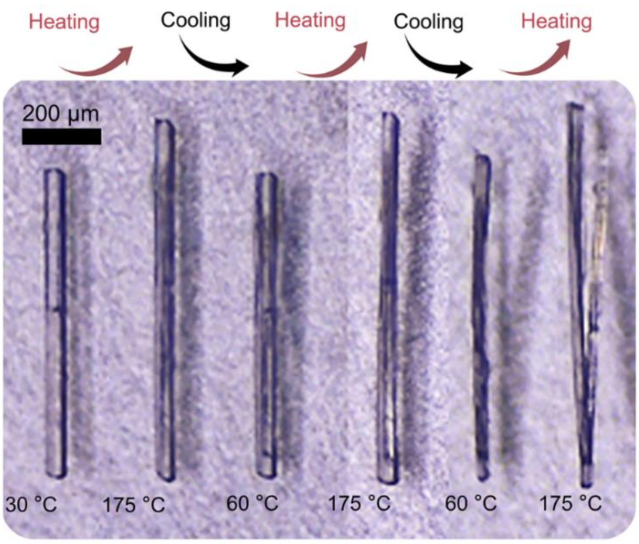

Figure S7. Reversibility in the elongation and shortening of an α-DBpT crystal upon repeated heating and cooling.

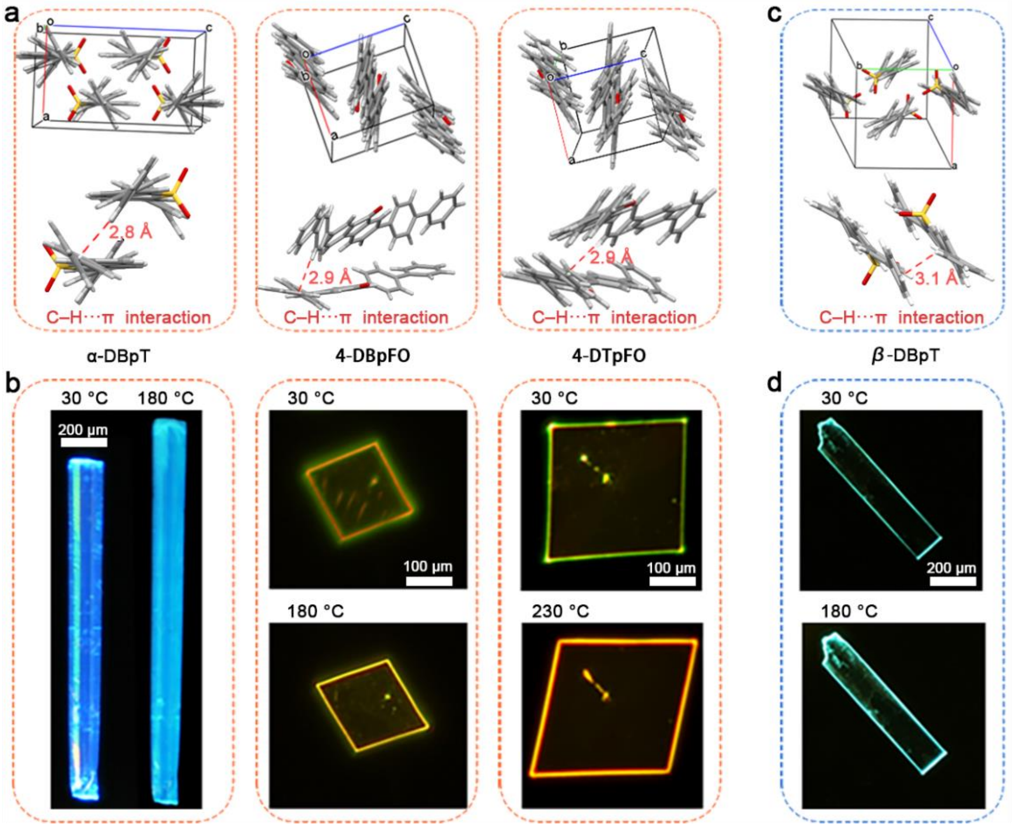

Figure S8. Morphology and crystal structures. (a) Packing structures and the nearest non-bonding interactions (C–H···π interaction) between neighboring molecules of α-DBpT, 4-DBpFO and 4-DTpFO crystals, and (b) their macroscopic morphology before and after the phase transition. (c) Packing structure and the nearest C–H···π interaction between neighboring molecules of β-DBpT crystal and (d) its crystal habit at high and low temperatures.

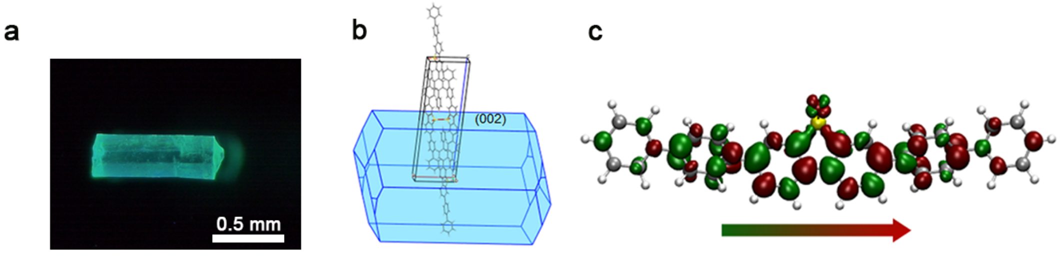

Figure S9. Optical properties of the β-DBpT crystal. (a) Fluorescent images of β-DBpT (λex = 365 nm). (b) Simulation of the morphology of the β-DBpT crystal. (c) The calculated transition charge density wave of β-DBpT molecule, indicating that the direction of transition dipole moment is nearly parallel to the long axis of the molecule of β-DBpT.

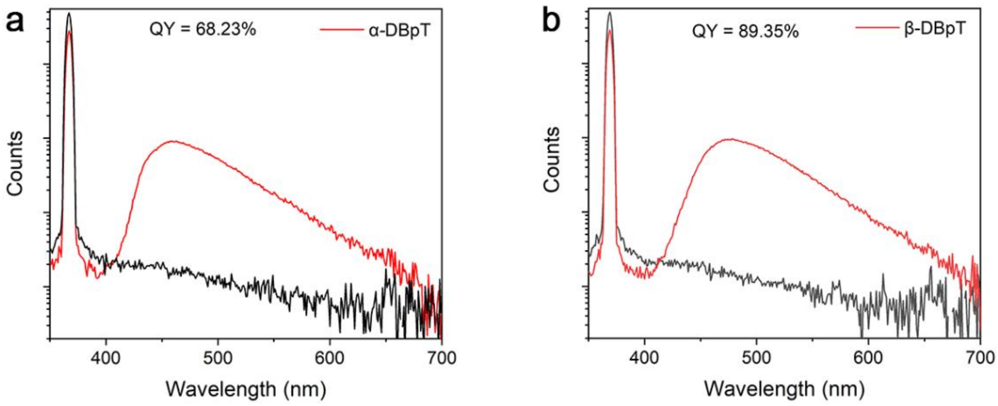

Figure S10. Photoluminescence spectra and quantum yields of (a) α-DBpT and (b) β-DBpT.

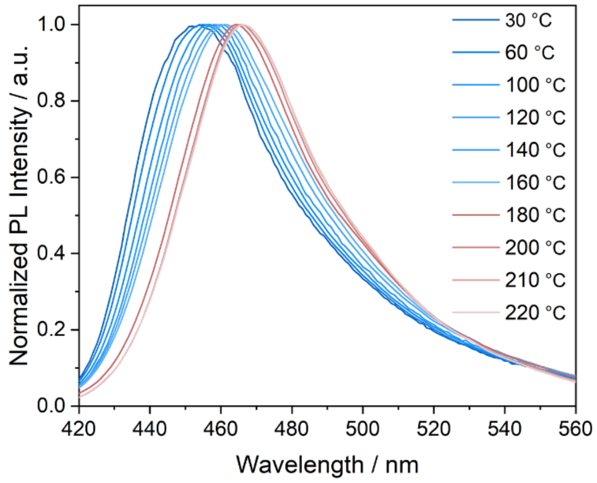

Figure S11. Normalized temperature-dependent fluorescence spectra of α-DBpT.

3. Supporting tables

Table S1. Linear expansion of α-DBpT crystals

**Initial length, L_0 (μm)**	**Length after heating, L (μm)**	**Elongation, ΔL (%)**
222.7	260.6	17.0
254.2	294.0	15.7
420.8	479.9	14.0
260.1	298.2	14.6
255.2	296.8	16.3
438.6	506.8	15.5
717.0	814.6	13.6
244.2	278.3	14.0
575.4	661.2	14.9
499.9	574.5	14.9
340.3	391.7	15.1
324.6	379.3	16.9
323.0	368.0	13.9
400.1	456.7	14.1
318.2	364.4	14.5
**Average elongation**		**15.0**

Table S2. Crystallographic and refinement data for DBpT

**Compound No.**	**α-DBpT (193 K)**	**α′-DBpT (470 K)**	**β-DBpT**
CCDC[#]	2286600	2286599	2286601
chemical formula		$C_{36}H_{24}O_2S$	
formula weight		520.61	
crystal system	monoclinic	orthorhombic	monoclinic
space group	$P2_1/c$	$Cmc2_1$	$P2_1/n$
a/Å	7.3	28.7	9.5
b/Å	28.8	11.0	9.3
c/Å	12.2	8.4	28.6
α/°	90	90	90
β/°	90.4	90	95.5
γ/°	90	90	90
V/Å^3	2569.47	2652.47	2518.73(5)
Z	4	4	4
ρ/(g cm^{-3})	1.346	1.304	1.373
F(000)	1088	1088	1092.563
R_1[a] [$I > 2\sigma(I)$]	0.0450	0.0708	0.0942
wR_2[b] [all data]	0.1274	0.1759	0.2628

[a]$R_1 = \Sigma||F_o| - |F_c|| / \Sigma|F_o|$. [b]$wR_2 = \{\Sigma[w(F_o^2 - F_c^2)^2] / \Sigma w(F_o^2)^2\}^{1/2}$.

Table S3. Summary of the data collected for nine individual crystals using the AE801 force sensor

Crystal	Length (m)	Width (m)	Thickness (m)	Volume (m^3)	Force (N)	Force Density (N m^{-3})	Stroke (m)	Work (J)	Work density (J m^{-3})
1	4.48E-03	1.15E-04	8.57E-05	4.42E-11	6.16E-06	1.39E+05	1.20E-05	7.39E-14	1.67E-03
2	1.35E-03	1.03E-04	9.83E-05	1.36E-11	2.24E-06	1.64E+05	9.15E-06	2.05E-14	1.50E-03
3	5.41E-04	1.33E-04	1.01E-04	7.27E-12	1.89E-07	2.60E+04	4.30E-06	8.13E-16	1.12E-04
4	8.66E-04	2.17E-04	1.97E-04	3.70E-11	3.19E-06	8.63E+04	8.10E-05	2.58E-13	6.99E-03
5	2.68E-03	1.03E-04	8.70E-04	2.40E-10	1.50E-05	6.26E+04	8.90E-05	1.34E-12	5.57E-03
6	1.37E-03	1.12E-04	1.00E-04	1.54E-11	1.98E-06	1.29E+05	7.60E-06	1.50E-14	9.78E-04
7	1.61E-03	1.20E-04	1.02E-04	1.98E-11	2.87E-06	1.45E+05	2.43E-05	6.98E-14	3.53E-03
8	1.36E-03	1.21E-04	1.00E-04	1.64E-11	4.74E-06	2.89E+05	9.25E-06	4.38E-14	2.67E-03
9	2.76E-03	1.30E-04	1.02E-04	3.65E-11	1.99E-06	5.46E+04	8.14E-06	1.62E-14	4.44E-04

4. Supporting references

(1) Sheldrick, G. M., SHELXT–Integrated space-group and crystal-structure determination. *Acta Crystallogr. A* **2015,** *71* (1), 3–8.

(2) Dolomanov, O.; Bourhis, L.; Gildea, R.; Howard, J.; Puschmann, H., Olex2—A complete package for molecular crystallography. *J. Appl. Crystallogr.* **2009,** *42*, 339–342.

(3) Khalil, A.; Karothu, D. P.; Naumov, P. Direct quantification of rapid and efficient single-stroke actuation by a martensitic transition in a thermosalient crystal. *J. Am. Chem. Soc.* **141**, 3371–3375 (2019).

(4) Ansys Granta Selector 2020, ver. R2, ANSYS, 2020. Website: https://www.ansys.com/products/materials/granta-selector

5. Legends for the supporting movies

Movie S1. Elongation of an α-DBpT crystal observed by using a light microscope. The initial length of the crystal was 575.4 μm, and after the phase transition, it was 661.2 μm, with elongation of ~15%.

Movies S2. Elongation an α-DBpT crystal recorded by using a high-speed camera. The video resolution is 1280 × 720 pixels, the frame rate of the high-speed camera is 4500 fps, and the length of the crystal is ~400 μm.

Movie S3. A movie showing a crystal that elongates and propels a glass sphere. The video resolution is 1280 × 720 pixels, the frame rate of the high-speed camera is 4500 fps, and the length of the crystal is ~500 μm.
